# Vaccinia virus-mediated intra-tumoral expression of matrix metalloproteinase 9 enhances oncolysis of PC-3 xenograft tumors

**DOI:** 10.1186/1471-2407-12-366

**Published:** 2012-08-23

**Authors:** Simon Schäfer, Stephanie Weibel, Ulrike Donat, Qian Zhang, Richard J Aguilar, Nanhai G Chen, Aladar A Szalay

**Affiliations:** 1 Department of Biochemistry, Biocenter, University of Würzburg, Würzburg, 97074, Germany; 2 Genelux Corporation, San Diego Science Center, San Diego, CA, 92109, USA; 3 Department of Radiation Oncology, Rebecca & John Moores Comprehensive Cancer Center, University of California San Diego, La Jolla, CA, 92093, USA

## Abstract

**Background:**

Oncolytic viruses, including vaccinia virus (VACV), are a promising alternative to classical mono-cancer treatment methods such as surgery, chemo- or radiotherapy. However, combined therapeutic modalities may be more effective than mono-therapies. In this study, we enhanced the effectiveness of oncolytic virotherapy by matrix metalloproteinase (MMP-9)-mediated degradation of proteins of the tumoral extracellular matrix (ECM), leading to increased viral distribution within the tumors.

**Methods:**

For this study, the oncolytic vaccinia virus GLV-1h255, containing the *mmp-9* gene, was constructed and used to treat PC-3 tumor-bearing mice, achieving an intra-tumoral over-expression of MMP-9. The intra-tumoral MMP-9 content was quantified by immunohistochemistry in tumor sections. Therapeutic efficacy of GLV-1h255 was evaluated by monitoring tumor growth kinetics and intra-tumoral virus titers. Microenvironmental changes mediated by the intra-tumoral MMP-9 over-expression were investigated by microscopic quantification of the collagen IV content, the blood vessel density (BVD) and the analysis of lymph node metastasis formation.

**Results:**

GLV-1h255-treatment of PC-3 tumors led to a significant over-expression of intra-tumoral MMP-9, accompanied by a marked decrease in collagen IV content in infected tumor areas, when compared to GLV-1h68-infected tumor areas. This led to considerably elevated virus titers in GLV-1h255 infected tumors, and to enhanced tumor regression. The analysis of the BVD, as well as the lumbar and renal lymph node volumes, revealed lower BVD and significantly smaller lymph nodes in both GLV-1h68- and GLV-1h255- injected mice compared to those injected with PBS, indicating that MMP-9 over-expression does not alter the metastasis-reducing effect of oncolytic VACV.

**Conclusions:**

Taken together, these results indicate that a GLV-1h255-mediated intra-tumoral over-expression of MMP-9 leads to a degradation of collagen IV, facilitating intra-tumoral viral dissemination, and resulting in accelerated tumor regression. We propose that approaches which enhance the oncolytic effect by increasing the intra-tumoral viral load, may be an effective way to improve therapeutic outcome.

## Background

Prostate cancer (PCa) is responsible for 11% of cancer-related deaths in men in the US, second only to lung and bronchial cancer cases [1]. In most patients, death is caused by the formation of metastases. Therefore, the success of therapy is strikingly dependent on the time point of diagnosis [2], and the efficacy of the therapy. Commonly used classical cancer therapies still show deficits in regard to their efficacy and/ or specificity. An exemplary obstacle for successful treatment is the development of resistance to chemo- or radiotherapy [3,4]. In the case of surgery, malignant cells can remain after the primary tumor is removed, which might lead to enhanced overall survival, but not a complete recovery [5].

Therefore, it is necessary to investigate alternative treatments. A promising therapeutic approach for the treatment of PCa are oncolytic viruses such as vaccinia virus (VACV) GLV-1h68 [6]. This virus was also successfully used in other malignancies such as pancreatic tumors, squamous cell carcinoma, breast cancer or in combination with a prodrug activated by the GLV-1h68-carried marker gene β-galactosidase [7-10]. VACV replicates exclusively in the cytoplasm [11] and has a large genome well suited for inserts of up to 25 kb [12]. This fact was exploited for the construction of further recombinant VACVs (rVACVs). The inserted genes can be used to enhance therapeutic efficacy as in the case of a GLAF-1 (a single chain antibody against vascular endothelial growth factor (VEGF)) encoding VACV [13], or to allow the use of imaging techniques such as positron emission tomography (PET) [14,15].

In this study, the possibility to use oncolytic viruses as a vector for the intra-tumoral expression of recombinant proteins was explored to further enhance virotherapy by elevating intra-tumoral titers, which may increase the oncolytic effect. Generally, the extracellular matrix (ECM) can be a hindrance for viral cell-to-cell spreading, as reported for oncolytic adenoviruses, decreasing the oncolytic effect. However, it was shown that the insertion of the *relaxin* gene in adenoviruses leads to the degradation of ECM components within the tumor microenvironment, thus improving viral spreading [16,17]. Further possible candidates, which can be used for the intra-tumoral degradation of ECM components, are the matrix-metalloproteinases (MMPs) [18]. MMPs are zinc-dependent endopeptidases, usually expressed as inactive zymogens. In their activated state, these MMPs are important for the breakdown of several ECM proteins, playing a role in various processes (e.g. tissue remodeling) and also in cancer progression [19,20]. In addition, the cleavage of ECM proteins can result in the shedding of ECM-bound growth factors, leading to multiple actions within the tumor microenvironment [21,22]. For example, not only has an enhanced angiogenesis been observed after MMP-mediated shedding of pro-angiogenic factors (e.g. VEGF), but also a decreased BVD due to anti-angiogenic factors (e.g. endostatin, tumstatin, angiostatin) was reported [23-25].

In this study, the oncolytic VACV GLV-1h255, containing the *mmp-9* gene, was constructed to specifically degrade the intra-tumoral ECM. Among the MMP-9 ECM substrates are collagen, laminin, fibrillin and elastin [26]. Indeed, we could observe a significantly decreased collagen IV content, accompanied by increased intra-tumoral virus titers in GLV-1h255colonized PC-3 tumors. Additionally, the tumor regression process started earlier in the treatment phase and was markedly accelerated. Our results suggest that a virus-mediated intra-tumoral degradation of ECM components offers a new strategy to optimize oncolytic rVACV tumor treatment.

## Methods

### Cell culture

PC-3 (DSMZ no. ACC465) and A549 (ATCC no. CCL-185) cells were cultured in RPMI 1640 supplemented with 10% fetal bovine serum (FBS) and antibiotic solution (100 units/mL penicillin G and 100 units/mL streptomycin) at 37°C and 5% CO_2_.

For CV-1 cells (provided by Genelux GmbH, Bernried) DMEM instead of RPMI 1640 was used. Media and additives were purchased from PAA (Pasching, Austria).

### Construction of recombinant VACV GLV-1h255

The human MMP-9 cDNA clone was originally from OriGene (Cat. SC116989), which encodes the *Homo sapiens* matrix metallopeptidase 9 (Accession No. NM_004994.2). The human MMP-9 cDNA was PCR amplified with primers MMP9-5 [5’-GTCGAC(Sal I) CACCATGAGCCTCTGGCAGCCC-3’] and MMP9-3 [5’-TTAATTAA(PacI) CTAGTCCTCAGGGCACTGCA-3’] with the above mentioned clone as the template. The PCR product was gel purified, and cloned into the pCR-Blunt II-TOPO vector using the Zero Blunt TOPO PCR Cloning Kit (Invitrogen). The resulting construct pCRII-MMP9-1 was sequence confirmed. The MMP9 cDNA was released from pCRII-MMP9-1 with Sal I and Pac I, and subcloned into the TK transfer vector with the same restriction sites, placing hMMP9 cDNA under the control of vaccinia early (SE) promotor. The resulting construct TK-SE-hMMP9 was sequence confirmed.

TK-SE-hMMP9 was used to generate the recombinant virus GLV-1h255, with GLV-1h68 as the parental virus, replacing the p7.5-*lacZ* expression cassette and pSEL-hTFR in the TK locus with the expression cassette of hMMP-9.

### MMP-9 protein analysis

PC-3 cells were infected with GLV-1h68 or GLV-1h255 at a multiplicity of infection (MOI) of 1. Supernatants were collected 24 h post infection (p.i.) and sterile-filtred (0.1 μm filter Schleicher & Schuell Dassel, Germany). Cells were washed once with PBS and lysed by addition of SDS sample buffer (130 mM Tris HCl pH 6.8, 4% (w/v) SDS, 20% (v/v) glycerol, 5.3% (v/v) 2-mercaptoethanol, 0.15 mM bromphenol blue). Supernatants were mixed (1:1) with SDS sample buffer. All samples were denatured, separated by SDS-PAGE and transferred to nitrocellulose membranes. MMP-9 was detected using a polyclonal anti-MMP-9 antibody produced in goat (Neuromics, Edina USA, GT15020) and a HRP-conjugated anti-goat antibody (Sigma, Steinheim Germany, A8919). ECL solution (90 mM p-coumaric acid, 250 mM luminol, 1 M Tris–HCl, pH 8.5) was used as a substrate for the HRP reaction. β-actin was detected using an anti-β-actin antibody produced in mouse (Abcam, Cambridge UK, ab6276) and a HRP-conjugated anti-mouse antibody (Abcam, Cambridge UK, ab6728).

For the zymography (Millipore, protocol MCPROTO 009, 2007), A549 cells were infected with GLV-1h68 or GLV-1h255 at an MOI 0.1 for 24 h. At this time point supernatants were collected and sterile-filtered. Cells were washed once with PBS and lysed by addition of non-reducing SDS sample buffer (w/o β-mercaptoethanol). Supernatants were mixed (1:1) with non-reducing SDS sample buffer. Protein samples were separated by SDS-PAGE using 10% SDS gels containing 1 mg/mL gelatin. After separation the proteins were renatured and developed for 1 d at 37°C. Gel staining was performed with 0.5% (w/v) Coomassie Brilliant Blue.

### Animal studies

PC-3 cells (2 × 10^6^ in 100 μL PBS) were implanted subcutaneously in the right abdominal flank of 6 – 8 week old female nude mice (NCl:Hsd:Athymic Nude *Foxn1*^*nu*^, Harlan Borchem, Germany). Tumors were measured weekly in two dimensions using a digital caliper and tumor volumes were calculated (length × width^2^/2). After approximately 3 weeks, a single dose of GLV-1h68 or GLV-1h255 (5 × 10^6^ plaque forming units (pfu) in 100 μL PBS) was injected into the tail vein (i.v.). Control animals were injected with 100 μL PBS. All animal experiments were carried out in accordance with protocols approved by the Regierung of Unterfranken (Würzburg, Germany, protocol number AZ 55.2-2531.01-17/08) and/ or the Institutional Animal Care and Use Committee (IACUC) of Explora BIOLABS, located in the San Diego Science Center (San Diego, USA) (protocol number: EB08-003).

### Viral titration in tumor samples

Tumors were snap-frozen in liquid nitrogen. After thawing tumors were weighed, cut into pieces using a scalpel and transferred to M-tubes (Miltenyi Biotec, Bergisch Gladbach, Germany) and mixed with the 2× volume of buffer (1 tablet of Roche complete mini proteinase inhibitor dissolved in 50 mL of PBS). Homogenization of tissues was done twice with a GentleMACS dissociator (Miltenyi Biotec). Subsequently, samples underwent 3 freeze/thaw cycles and were sonified 3 times for 30 s each time. Virus titers were obtained by performing a standard plaque assay on CV-1 cells.

### Virus replication assay

To analyze the *in vitro* growth kinetics of GLV-1h68 and GLV-1h255, PC-3 cells were seeded in 24 well plates. When the cell layer reached 90% confluency, cells were infected with an MOI of 0.1 in triplicates for each time point. At 1, 2, 4, 6, 10, 24, 48 and 72 hours post infection supernatants were obtained and cells were washed with Hank’s BSS (PAA, Pasching, Austria). Cells were detached using trypsin and pelleted by centrifugation and resuspended in Hank’s BSS. All samples were snap frozen in liquid nitrogen and stored at −80°C. To obtain the viral titers, a standard plaque assay on CV-1 cells was performed. Prior to the plaque assay, three freeze-and-thaw cycles were completed to achieve a thorough cell lysis.

### Analysis of lumbar and renal lymph nodes

For the analysis of lymph node metastases in PC-3 tumor bearing mice, mice were sacrificed 24 days after virus treatment as described above. To calculate the volumes of lumbar and renal lymph node metastases, the abdomen was opened and all organs were removed. A picture of the now visible lymph nodes was taken, using a ruler as a guide. The width and length were measured using Photoshop CS4 (Adobe Systems, Mountain View, CA). Lymph node volumes were calculated using the formula: length × width^2^/2.

### Histology, fluorescence microscopy and image analysis

100 μm tumor sections were prepared as described previously [27]. All of the following steps were carried out at room temperature. Tissue sections were permeabilized by incubation in PBS containing 0.2% Triton X-100 and 5% FBS (blocking solution) for 1 h. For fluorescence labeling, sections were incubated for 12 h with the primary antibody in blocking solution. Subsequently, sections were washed with PBS and incubated with the corresponding secondary antibody in blocking solution for 4 h. Finally, tumor sections were washed in PBS and mounted onto glass slides using Mowiol/DABCO.

The fluorescence labeled specimens were examined using a stereo-fluorescence microscope (MZ16 FA, Leica) equipped with a color CCD camera (DC500, Leica). Images were acquired using the Leica IM1000 v 4.0 software and Photoshop CS4 was employed to adjust levels and create overlays.

Collagen IV in PC-3 tumor sections was labeled with an anti-collagen IV antibody (Abcam, Cambridge UK, ab19808). Quantification was performed using ImageJ ( http://rsbweb.nih.gov/ij/). Three images (100x, Leica MZ16 FA) from infected and three images from non-infected areas were taken from each section. Images taken from 3 non-consecutive sections for each tumor, and 3 tumors per group were used for analysis.

MMP-9 expression was analyzed in PC-3 tumor sections labeled with MMP-9 antibody (Neuromics, Edina USA, GT15020). Image acquisition was done as described before, except that a magnification of 150x was used (Leica MZ16 FA). Visualization of MMP-9 expression was also done with these sections. All images were converted from RGB to grayscale for ImageJ analysis (Photoshop CS4), with mean fluorescence intensity measurements reported.

### Analysis of blood vessel density

The blood vessel density was determined using microscopic images (200x, Leica MZ16 FA) of CD31-labeled (CD31 antibody, Millipore, mab1398Z) tumor sections. Three non-consecutive sections from each tumor were taken and 3 tumors per group were analyzed. For each section, 3 images from infected and 3 from non-infected areas were taken. Exposure time was adjusted to ensure clear visibility of the blood vessels in each section. Subsequently, 5 equidistant lines were drawn in a template using Adobe Illustrator CS4. Each individual image was superimposed with the template and vessels crossing the lines were counted. The average blood vessel density per section with standard deviation is shown.

### Statistical analysis

A two-tailed Student’s *t* test was used to determine statistical significance (*p* values: * *p* < 0.05, ** *p* < 0.01, *** *p* < 0.001). Error bars represent standard deviation.

## Results

### MMP-9 is expressed in GLV-1h255 infected PC-3-cells and shows enzymatic activity *in vitro*

**Figure 1:**
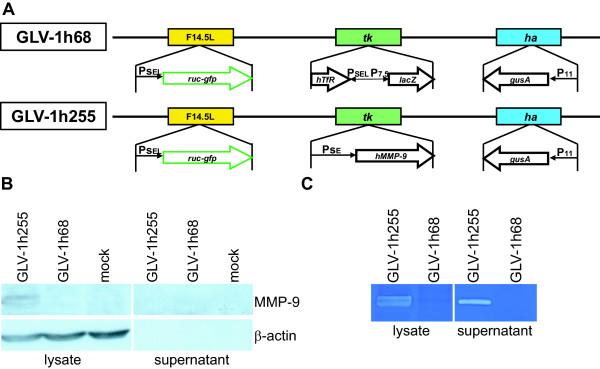
**Expression of functional MMP-9 by GLV-1h255-infected tumor cells.** (**A**) Expression cassettes of GLV-1h68 and GLV-1h255. In GLV-1h255 the insert in the Tk locus was replaced by the human *mmp-9* gene under control of the P_SE_ promoter. P_SEL,_ synthetic early/late promoter; P_SE,_ synthetic early promoter; P_7.5,_ VACV p7.5 K early/late promoter_;_ P_11,_ VACV p11 late promoter; Tk, thymidine kinase locus, Ha, hemagglutinin locus. (**B**) Expression of virus-encoded MMP-9 (92 kDa) in GLV-1h255 infected PC-3 cells and supernatants *in vitro*, β-actin (42 kDa) was used as a loading control. (**C**) Activity of the MMP-9 protein was tested by gelatin zymography. Lysates and supernatants of infected A549 cells were isolated and separated by non-reducing SDS-PAGE. In zymography, cleavage of the substrate by MMP-9 resulted in a clear band.

In this study, the rVACV GLV-1h255 was constructed to express human MMP-9 under control of the synthetic early promoter as shown in (Figure 1A). The expression of MMP-9 by GLV-1h255 infected PC-3 cells was confirmed by Western Blot (Figure 1B). To investigate enzymatic activity of secreted MMP-9 by zymography, we infected A549 lung carcinoma cells with either GLV-1h68 or GLV-1h255. In A549 cells, which are well suited for protein over-expression, abundant protein expression allowed the visualization of enzymatic activity of MMP-9 in both cell-lysates and the sterile-filtered supernatants (Figure 1C).

### MMP-9 is expressed in GLV-1h255 infected areas of PC-3 tumors

**Figure 2:**
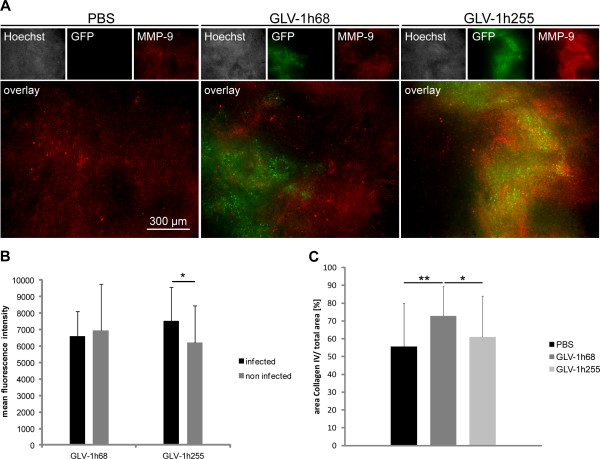
**MMP-9 expression in PC-3 tumor sections and collagen IV quantification.** Intratumoral expression of MMP-9 (red) was visualized using MMP-9 labeled PC-3 tumor sections. Nuclei (white) were stained with Hoechst 33258 dye. GFP (green) is a VACV reporter gene. Tumors were obtained at 7 days p.i. from PC-3 tumor-bearing mice injected with PBS, GLV-1h68 or GLV-1h255. All images are representative examples. (**B**) Quantification of MMP-9 expression was done by microscopic analysis. Mean fluorescence intensities were measured with ImageJ. (**C**) Quantification of collagen IV 7 days p.i. in GLV-1h68 or GLV-1h255 infected areas of PC-3 tumor sections. Images were taken at a 100× magnification (Leica MZ16 FA) and converted from RGB to grayscale using Photoshop. For image analysis ImageJ was used, the threshold value was 8/255.

Expression of MMP-9 was also analyzed in PC-3 tumor sections at 7 days p.i. In PC-3 tumors from either PBS or GLV-1h68 treated animals, an already high basal expression level of MMP-9 was detected, probably associated with immune cells. Nonetheless, a specific MMP-9 over-expression was observed in GLV-1h255 infected tumors, compared to tumors of both control groups (Figure 2A). The over-expression of MMP-9 co-localized with GFP-positive GLV-1h255-infected tumor areas. In addition, the MMP-9 expression was significantly higher in GLV-1h255 infected areas compared to non-infected areas (Figure 2B). In comparison, MMP-9 expression in GLV-1h68 infected areas was considerably lower than in GLV-1h255 infected areas.

### Virus-mediated MMP-9 expression enhances collagen IV degradation in GLV-1h255 infected tumor areas

To analyze whether MMP-9 activity in GLV-1h255 infected tumors leads to the degradation of ECM components, we investigated the distribution of collagen IV, a protein of the ECM and substrate of MMP-9, in tumors. Indeed, in infected areas of GLV-1h255 infected tumors, the collagen IV content was significantly lower than in GLV-1h68 infected areas (Figure 2C). In general, however, we observed a significantly higher collagen IV content in GLV-1h68 infected tumors in comparison to PBS treated tumors. This increase might be due to the viral infection, leading to an uncontrolled inflammation in the tumor microenvironment (reviewed in [28]), which may result in a pronounced fibrotic deposition of collagen. Therefore, virus-mediated MMP-9 expression might minimize infection-related collagen IV fibrosis.

### GLV-1h255 treatment of tumors accelerates the tumor regression process by increasing intra-tumoral virus titers

**Figure 3:**
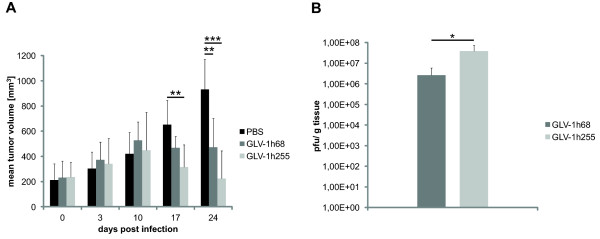
**Tumor regression analysis and virus titers in GLV-1h68 and GLV-1h255 infected tumors.** (**A**) Growth of PC-3 xenograft tumors in athymic nude mice. Animals were i.v. injected with GLV-1h68, GLV-1h255 or PBS. Tumor volumes were measured weekly (n = 6–7). Statistical significance was determined by using a two-tailed Student’s *t* test (*p*-values: * < 0.05, ** < 0.01, *** < 0.001). (**B**) Viral titers (pfu/g tissue) of tumor homogenates 7 days p.i. Titers were obtained by performing a plaque assay on CV-1 cells. Four tumors per group (GLV-1h68, GLV-1h255) were used.

To determine whether the virus-mediated expression of MMP-9 and degradation of collagen IV may improve oncolytic viral therapy, PC-3 tumor-bearing mice were injected with a single dose of GLV-1h255. Control animals were injected with either GLV-1h68 or PBS. The three phased growth pattern (growth, inhibition, regression) of PC-3 xenograft tumors after viral injection was in accordance with previous observations [10]. Significant tumor regression already occurred at 17 days p.i. in GLV-1h255 treated animals, but not in those treated with GLV-1h68. This indicates an enhanced oncolytic effect of GLV-1h255, compared to the parental GLV-1h68 virus. Furthermore, tumors of GLV-1h255 injected animals showed a significantly enhanced regression at 24 days p.i. when compared with PBS injected mice (*p* < 0.001), whereas the difference in volumes between GLV-1h68 and PBS treated tumors was also significant, but to a lesser extend (*p* < 0.01) (Figure 3A).

**Figure 4:**
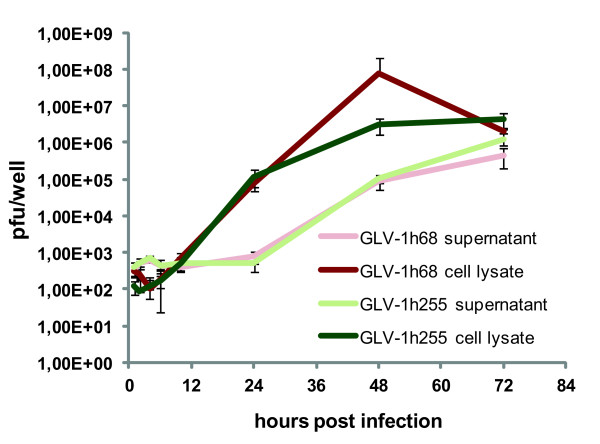
**Replication kinetics of GLV-1h68 and GLV-1h255 in PC-3 cells.** PC-3 cells were infected with GLV-1h68 and GLV-1h255 with an MOI of 0.1. Virus titers of infected PC-3 cells and their supernatants were determined 1, 2, 4, 6, 10, 24, 48 and 72 hours post infection by a standard plaque assay using CV-1 cells. Triplicates of infected PC-3 cells were used for each time point.

Since the primary idea for the construction of GLV-1h255 was to increase viral spreading and viral load in infected tumors, enhancing the oncolytic effect, we analyzed virus titers in GLV-1h68 and GLV-1h255 infected tumors at 7 days p.i. by plaque assay. Indeed, at this early time point after infection, GLV-1h255 infected tumors showed a significantly higher average virus titer than those infected with GLV-1h68 (Figure 3B). To exclude the possibility that the higher titers were due to a better replication of GLV-1h255, *in vitro* replication in PC-3 cells was analyzed by a replication assay (Figure 4). In this case the replication of GLV-1h255 was not enhanced when compared to the replication of GLV-1h68.

### Over-expression of MMP-9 in GLV-1h255 infected tumors does not lead to an enhanced angiogenesis

**Figure 5:**
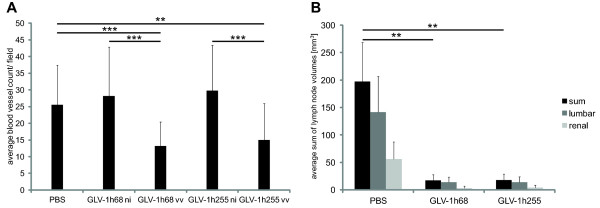
**Analysis of lumbar and renal lymph node metastases in PC-3 tumor-bearing mice and of Here, the blood vessel density should be mentioned first (Figure**5A**, text labeled in yellow) and lymph nodes second (Figure**5B**).** (**A**) Blood vessel density in PC-3 tumor sections, 7 days p.i. For GLV-1h68 and GLV-1h255 blood vessels in infected (vv) and non-infected (ni) areas were counted. Infected areas were determined by expression of the viral marker GFP. For each image (200x, Leica MZ16 FA) blood vessels crossing 5 equidistant lines were counted. (**B**) Analysis of renal and lumbar lymph node enlargement. PC-3 tumor-bearing mice were injected i.v. with GLV-1h68, GLV-1h255 or PBS. The mice were sacrificed 24 days p.i. and lymph nodes were measured (n = 6–7).

Angiogenesis is often linked to elevated expression levels of MMP-9 as it influences the bioavailability of VEGF [29]. Therefore, intra-tumoral over-expression of MMP-9 could theoretically be associated with enhanced angiogenesis. We determined the vascular density in PC-3 tumor sections at 7 days p.i. to elucidate whether it is influenced by the GLV-1h255 mediated over-expression of MMP-9 (Figure 5A). In GLV-1h68 or GLV-1h255 infected areas of tumors, the vascular density was significantly lower than in uninfected areas or sections from tumors of PBS injected animals. Additionally, there was no significant difference in the BVD between GLV-1h68- and GLV-1h255-infected tumor areas. Thus, GLV-1h255 treatment of PC-3 tumor-bearing mice did not enhance angiogenesis.

### GLV-1h68 and GLV-1h255 have a strong therapeutic effect on renal and lumbar lymph node metastases in PC-3 tumor-bearing mice

PC-3 xenograft tumors have a high metastatic potential in athymic nude mice [30-32] and the expression of ECM-degrading enzymes might additionally increase the shedding of tumor cells, resulting in elevated metastasis formation. Of these metastases, the renal and lumbar ones can be easily analyzed after a removal of organs from the abdomen. To determine whether the intra-tumoral expression of MMP-9 in GLV-1h255 treated PC-3 tumors influence the metastatic spread, the volume of renal and lumbar lymph nodes was measured 24 days p.i. (Figure 5B). The renal and lumbar lymph nodes in GLV-1h255 injected mice had similar volumes as in GLV-1h68 treated mice, and both were significantly smaller than in PBS injected ones. Therefore, the virus-mediated over-expression of MMP-9 enhances virotherapy of the primary tumor while sustaining the rVACV-metastasis reducing effect.

## Discussion

The use of oncolytic VACV is a promising approach for the treatment of various types of cancer [7-10]. However, the therapeutic efficacy relies extensively on the efficient oncolysis of tumor cells [27], indicating that an efficient viral spreading inside tumor mass is essential. In this context, proteins of the ECM have been identified as an obstacle for the intra-tumoral spreading of virus particles [33,34].

Two recently published studies reported that adenovirus-mediated intra-tumoral expression of relaxin resulted in an increased tumor regression [16,17].

In the present study, we showed for the first time that degradation of the tumoral ECM also enhanced therapeutic efficacy of oncolytic rVACV. To achieve tumoral ECM degradation, we used GLV-1h255 encoding *mmp-9*, for the treatment of PC-3 tumor-bearing mice.

PC-3 tumors of GLV-1h68 and PBS injected mice showed a high basal MMP-9 expression. This might be due to the presence of non-malignant stromal cells, e.g. inflammatory cells such as neutrophils, macrophages or lymphocytes, most of which express MMP-9 [22]. Since PC-3 xenograft tumors have a high metastatic potential [30-32] and the degradation of ECM proteins by MMP-9 may facilitate metastasis [21], we analyzed the size of lumbar and renal lymph nodes in PC-3 tumor bearing mice, as these have been shown to be colonized by tumor cells, and increased in size compared to healthy ones [6]. However, the GLV-1h255 mediated over-expression of MMP-9 did not further enlarge these lymph nodes, as they had a similar size compared to GLV-1h68-treated animals and were significantly smaller than those in PBS treated animals. In accordance, Lavilla-Alonso *et al*. reported no increased tumor invasiveness by macrophage metalloelastase, another ECM degrading enzyme, when administered in combination with an oncolytic virus [35].

As MMP-9 can also induce angiogenesis by increasing the bioavailability of VEGF [29] which may stimulate primary tumor growth, we quantified the BVD in sections of tumors from infected and uninfected mice. The infected areas of both GLV-1h68- and GLV-1h255 treated tumors revealed similar BVD, which was significantly reduced compared to PBS-treated tumors.

Taken together, neither an increase in size of renal and lumbar lymph nodes, nor an induction of angiogenesis was observed in PC-3 tumor-bearing mice injected with GLV-1h255. Therefore, the final biological function of MMP-9 may strongly depend on the context and the local microenvironment of MMP-9 over-expression.

In this study, we propose that the enhanced oncolytic effect of GLV-1h255 could be due to the MMP-9-mediated collagen IV degradation in PC-3 tumors.

Interestingly, the collagen IV content was significantly increased in GLV-1h68 infected tumors compared to PBS-treated tumors, which might be a result of an inflammatory reaction caused by viral infection. It has already been reported that tumor-targeted microbes, such as bacteria and viruses [27,36], induce an overwhelming inflammatory reaction in the tumor microenvironment, which is accompanied by an uncontrolled collagen deposition. In normal wounded tissue, inflammation is tightly controlled and ultimately leads to scar formation and healing. Tumors however, which also have been described by Dvorak as “wounds that do not heal” [37], do not provide an orderly environment for the resolution of an infection focus [28]. This may explain the excessive deposition of collagen IV in the context of infection. The significantly decreased collagen IV content in GLV-1h255 infected areas may be the reason for the significantly higher virus-titer in GLV-1h255 infected tumors, compared to those infected with GLV-1h68, due to an accelerated extracellular cell to cell spreading. This would be in line with the findings of Ganesh *et al*. and Kim *et al*., who reported an increased tumor infection by adenoviruses after the degradation of ECM proteins [16,17]. This may furthermore explain the accelerated regression of GLV-1h255 infected tumors, as higher titers could result in an increased oncolysis, which has been shown to be an important factor for the therapeutic efficacy of VACV [27].

Collectively, our study revealed that the degradation of ECM within the tumor microenvironment can boost the oncolytic effect of rVACV. Moreover, we showed here that high levels of local, intra-tumoral ECM-degrading enzymes can be produced by virus-infected tumor cells themselves, certainly limiting systemic side effects.

## Conclusions

In summary, the present study revealed that the degradation of collagen IV (ECM) by VACV-encoded MMP-9 may represent a new option to significantly enhance the oncolytic effect of rVACV in PC-3 xenografts. We confirmed that the degradation of collagen IV facilitated viral infection of the tumor tissue, represented by significantly higher viral tumor titers and an accelerated tumor regression. Furthermore, both oncolytic viruses, parental GLV-1h68 and *mmp-9*-encoding GLV-1h255, significantly reduced the size of lumbar and renal lymph node metastases, indicating that MMP-9 enhances both virotherapy of the primary tumor and sustains the rVACV-metastasis reducing effect.

## Competing interests

This work was supported by grants from Genelux Corporation (R&D facility in San Diego, CA, USA). SW and UD received a postdoctoral fellowship, SS received a graduate fellowship awarded to the University of Würzburg, Germany by Genelux Corporation. QZ, RJA, NGC and AAS are employees of Genelux Corporation and have financial interests in Genelux Corporation.

## Authors’ contributions

SS conceived and designed the study, performed experiments, analyzed data and wrote the manuscript. SW helped to analyze tumor sections and helped to draft the paper. UD helped to analyze lymph nodes. UD also performed the in vitro virus replication assay. QZ, RJA and NGC constructed the recombinant vaccinia virus. AAS conceived the study and participated in coordination and drafting of the manuscript. All authors read and approved the final manuscript.

## Pre-publication history

The pre-publication history for this paper can be accessed here:

http://www.biomedcentral.com/1471-2407/12/366/prepub
